# Dietary Nutrient Intake and Sleep

**DOI:** 10.3390/nu15102276

**Published:** 2023-05-11

**Authors:** Georgia Trakada

**Affiliations:** Division of Pulmonology, Department of Clinical Therapeutics, School of Medicine, National and Kapodistrian University of Athens, 11528 Athens, Greece; gtrakada@hotmail.com

Various hormones and neuropeptides implicated in energy metabolism also regulate sleep cycles and wakefulness and promote adequate and restorative sleep. A bidirectional relationship exists between eating and sleeping; wakefulness is usually linked to hunger, whereas somnolence often coexists with satiety.

Sleep is regulated by circadian rhythms, sleep–wake homeostasis, and working or social norms. Circadian and ultradian patterns also regulate food intake; however, meal times, size, and composition are primarily a part of a daily routine, related to individual habits and social norms. Specific dietary components (macronutrients, micronutrients, and whole foods), different diets (Mediterranean, vegetarian, vegan), or different meal times (meals near bedtime, fasting, Ramadan) could influence sleep in terms of duration, quality, and sleep–wake schedules.

Jansen EC et al. [1] found in a cohort of 559 young children from north and north-central Appalachia, that inconsistent mealtimes, higher milk consumption, and lower fruit and vegetable consumption were each associated with shorter nighttime sleep duration trajectories over time. Ramón-Arbués, E et al. [2] revealed that among 868 Spanish students, unhealthy eaters were more likely to also be bad sleepers; an imbalanced intake of vegetables, fruits, dairy products, lean meats, legumes, sweets, and sugary soft drinks was associated with lower sleep quality. Saidi, O et al. [3] depicted in a crossover study of 24 healthy men, that increased carbohydrate intake was associated with better sleep quality and increased melatonin secretion. The protocol design included two sessions of three days on isocaloric diets; high-protein, low-carbohydrate (HPLC), or low-protein, high-carbohydrate (LPHC) followed by 24 h free living assessments. Additionally, McDonald BW and Watson PE [4] demonstrated that subjective sleeping difficulties in 370 women in the second and 310 women in the third trimester of pregnancy increased with the week of gestation, morning sickness severity, anxiety, dairy, and saturated fat intake, and decreased with fruit, vegetable, and monounsaturated fat intake.

Further, sleep habits exert a great influence on food intake parameters. According to a review analysis by Rusu A et al. [5], variability in sleep timing, either as social jet lag (SJL) or day-to-day variability, promoted an unhealthy diet characterized by lower consumption of fruits, vegetables, whole grains, beans, and a higher intake of sugar and meat. Persons with SJL often had a higher perceived appetite for energy-dense foods, delayed mealtime, and eating jetlag. However, limited evidence existed to verify an association between these characteristics and increased caloric intake or change in macronutrient intake.

Finally, both sleep and feeding behavior contribute to cardiovascular (CVD) health. The coexistence of obstructive sleep apnea (OSA)—which is characterized by repetitive arousals from sleep due to respiratory pauses and non-restorative sleep—and diabetes mellitus type 2 (T2DM)—which is characterized by metabolic dysregulation—synergistic increase CVD risk and deteriorate patients’ prognosis [6]. Targeted therapy for OSA and T2DM seems to substantially improve CVD prognosis and metformin, the cornerstone in the management of T2DM, may improve sleep quality, although it does not modify the prevalence of OSA.

Sleep and diet are crucial in growth, maturation, health, and energy balance. In this Special Issue, we welcomed articles concerning the impact of diet on sleep and vice versa, as a better understanding of possible interactions between them could improve both public health and clinical practice. With this information, we can recommend individualized suggestions about eating and sleeping better to minimize cardiometabolic risk and live a healthier life.
